# Real-World Toxicity and Hospitalization Outcomes Following Immunotherapy for Malignant Pleural Mesothelioma

**DOI:** 10.7759/cureus.119661

**Published:** 2026-11-14

**Authors:** Reshma David, Rehmatullah Khan

**Affiliations:** 1 Acute Medicine, Queen Elizabeth Hospital Birmingham, University Hospitals Birmingham NHS Foundation Trust, Birmingham, GBR; 2 Oncology, Queen Elizabeth Hospital Birmingham, University Hospitals Birmingham NHS Foundation Trust, Birmingham, GBR

**Keywords:** cancer immunotherapy, hospitalization, malignant pleural mesothelioma (mpm), overall survival, progression-free survival (pfs)

## Abstract

Introduction

Malignant pleural mesothelioma (MPM) is an aggressive disease with poor outcomes despite advances in systemic treatment. Combination immune checkpoint inhibitor therapy with nivolumab and ipilimumab is increasingly used in clinical practice for advanced MPM; however, real-world treatment outcomes and treatment-related toxicities remain important considerations. This study evaluated survival outcomes, treatment response, toxicity, and hospitalization in patients with advanced mesothelioma receiving combination immunotherapy with nivolumab and ipilimumab.

Methods

We conducted a retrospective cohort study of 24 patients with advanced mesothelioma who received nivolumab and ipilimumab at a tertiary NHS hospital in Birmingham, United Kingdom, between April 2021 and October 2025. Demographic and clinical characteristics, treatment exposure, radiological response, progression-free survival (PFS), overall survival (OS), adverse events, and hospital admissions were reviewed. Survival outcomes were assessed using Kaplan-Meier analysis.

Results

Twenty-four patients were included, of whom 23 (95.8%) had pleural disease and 14 (58.3%) had an Eastern Cooperative Oncology Group performance status of 1. The median number of treatment cycles was five (range, 1-24). Recorded response categories were progressive disease in 12 patients (50%), stable disease in seven (29.2%), partial response in three (12.5%), and mixed response in two (8.3%). Median PFS was 4.5 months, and median OS was 9.5 months. Grade 3 or higher immune-related adverse events occurred in six patients (25%). Twenty patients (83.3%) experienced at least one hospital admission, with 36 admission episodes recorded. Admissions were attributed to immunotherapy-related adverse effects in 16 patients and disease-related or other non-immune complications in four patients.

Conclusions

Outcomes following nivolumab and ipilimumab varied considerably in this real-world cohort. Hospital admissions occurred in a substantial proportion of patients and were attributed to both immunotherapy-related adverse effects and disease-related complications. These findings highlight the importance of careful assessment, toxicity recognition, and multidisciplinary management during treatment.

## Introduction

Malignant pleural mesothelioma (MPM) is a rare and aggressive malignancy arising from the pleural lining and is strongly associated with previous asbestos exposure. Most patients present with advanced or unresectable disease, and prognosis remains poor despite improvements in supportive and systemic treatment [1]. For many years, platinum-based chemotherapy combined with pemetrexed was the standard first-line systemic treatment for patients with unresectable MPM. Although this approach provides clinical benefit, treatment outcomes remain limited, and there is considerable variation between patients [2].

The development of immune checkpoint inhibitors has changed the treatment landscape for MPM. In the phase III CheckMate 743 trial, first-line nivolumab combined with ipilimumab significantly improved overall survival (OS) compared with platinum-based chemotherapy in patients with unresectable MPM [3]. The benefit was observed across histological subgroups, supporting the use of dual immunotherapy as an important treatment option for unresectable MPM [3]. Current clinical guidance recognizes nivolumab plus ipilimumab as a first-line treatment option, although treatment selection continues to depend on factors such as histology, performance status, comorbidities, and individual patient circumstances [1].

While clinical trials provide important evidence for the efficacy and safety of immunotherapy, outcomes in routine clinical practice may differ because patients treated in the real-world setting can have different clinical characteristics, comorbidities, and treatment experiences [4-6]. In addition to survival and radiological response, treatment-related toxicity and hospitalization are clinically important outcomes that may affect treatment continuation and overall patient care [4,5]. Real-world data can therefore provide useful information about the effectiveness and tolerability of immunotherapy outside the clinical trial setting [4-7].

The aim of this study was to evaluate real-world outcomes in patients with advanced mesothelioma receiving immunotherapy. We assessed OS, progression-free survival (PFS), treatment response, treatment-related toxicity, and hospitalization burden. We also described baseline patient characteristics and treatment exposure to provide a better understanding of immunotherapy outcomes in routine clinical practice.

This study was previously presented as a poster at the 24th British Thoracic Oncology Group (BTOG) Annual Conference in Edinburgh, United Kingdom, from March 4 to 6, 2026.

## Materials and methods

Study design and setting

This retrospective cohort study was conducted at a large tertiary university teaching hospital in Birmingham, United Kingdom. Clinical records were reviewed for patients with advanced mesothelioma who received combination immunotherapy with nivolumab and ipilimumab between April 2021 and October 2025.

Study population

All eligible patients with advanced mesothelioma who received nivolumab and ipilimumab during the study period were included consecutively. A total of 24 patients were identified. The cohort included patients with both pleural and peritoneal disease. No eligible patients were excluded from the study.

Data collection

Data were retrospectively collected from electronic clinical records. Baseline patient characteristics, including age, ethnicity, Eastern Cooperative Oncology Group (ECOG) performance status, histological subtype, and primary disease site, were recorded. Other variables included treatment duration and number of treatment cycles, radiological assessment, disease response, progression, survival status, treatment-related toxicities, and hospital admissions occurring during or shortly after treatment with nivolumab and ipilimumab. Hospitalization data included the number of patients admitted, the total number of admission episodes, documented causes of admission, and length of hospital stay.

Treatment response and survival outcomes

Treatment response was assessed retrospectively in all 24 patients using available radiological imaging and clinical documentation during treatment. Where sufficient imaging information was available, radiological response was evaluated according to the Response Evaluation Criteria in Solid Tumors (RECIST). Responses documented in the clinical records were categorized as progressive disease, stable disease, partial response, or mixed response, where applicable.

PFS was defined as the time from initiation of nivolumab and ipilimumab to documented disease progression or death from any cause, whichever occurred first. Patients who had not experienced disease progression or death by the end of the follow-up period were censored at their last documented disease assessment. OS was defined as the time from initiation of nivolumab and ipilimumab to death from any cause. Patients who were alive at the end of the follow-up period were censored at the date of their last documented follow-up.

Assessment of treatment-related toxicity

Adverse events were identified from the clinical records and graded according to the Common Terminology Criteria for Adverse Events (CTCAE), version 5.0 [8]. Toxicities were categorized according to the affected organ system. Particular attention was given to hematological, gastrointestinal, endocrine, hepatic, respiratory, renal, dermatological, and musculoskeletal adverse events, as immune checkpoint inhibitors can cause a broad range of organ-specific toxicities affecting these systems [7]. These categories were used to capture the range of adverse events documented in the study cohort.

Immune-related adverse events (irAEs) were distinguished from other adverse events according to their documented clinical attribution. Events occurring during immunotherapy were not automatically classified as treatment-related, particularly where underlying malignancy, comorbidities, or other clinical causes provided an alternative explanation. The occurrence and severity of irAEs, treatment discontinuation due to toxicity, and hospital admissions associated with treatment complications were also recorded.

Statistical analysis

Descriptive statistics were used to summarize baseline characteristics, treatment exposure, treatment response, survival outcomes, toxicities, and hospitalizations. Median PFS and OS were estimated using the Kaplan-Meier method. Given the small sample size and retrospective nature of the study, the analysis was primarily descriptive, and no formal inferential statistical testing was performed.

Ethical approval

This study was conducted as a clinical audit and registered with the Clinical Audit Department of the participating NHS Trust. As the project was classified as a clinical audit, formal NHS Research Ethics Committee approval was not required. Patient confidentiality was maintained throughout data collection, analysis, and reporting in accordance with institutional policies and information governance requirements.

## Results

Baseline characteristics

A total of 24 patients with advanced mesothelioma received nivolumab and ipilimumab between April 2021 and October 2025. The majority had an ECOG performance status of 1, epithelioid histology, and pleural disease. Most patients were White British. Baseline demographic and clinical characteristics of the study cohort are summarized in Table 1.

**Table 1:** Baseline characteristics of the study cohort. Data are presented as n (%). ECOG, Eastern Cooperative Oncology Group

Characteristic	n (%)
Total patients	24 (100)
ECOG performance status	
ECOG 0	10 (41.7)
ECOG 1	14 (58.3)
Histology	
Epithelioid	19 (79.2)
Sarcomatoid	4 (16.7)
Biphasic	1 (4.2)
Primary disease site	
Pleural	23 (95.8)
Peritoneal	1 (4.2)
Ethnicity	
White British	20 (83.3%)
Black	2 (8.3%)
Other ethnicity	2 (8.3%)

Treatment exposure and response

The number of treatment cycles ranged from one to 24, with a median of five cycles. Treatment response was assessed in all 24 patients using available radiological imaging and clinical documentation. Seven patients (29.2%) had stable disease, three (12.5%) had a partial response, and two (8.3%) had a mixed response. The remaining 12 patients (50%) had progressive disease. Overall, disease control was documented in 12 patients (50%). Treatment exposure, response, toxicity, and hospitalization outcomes are summarized in Table 2.

**Table 2:** Treatment exposure, response, toxicity, and hospitalization outcomes. Data are presented as n (%) unless otherwise stated. irAE, immune-related adverse event; OS, overall survival; PFS, progression-free survival

Outcome	Result
Median number of treatment cycles	5
Range of treatment cycles	1-24
Progressive disease	12 (50%)
Stable disease	7 (29.2%)
Partial response	3 (12.5%)
Mixed response	2 (8.3%)
Median PFS	4.5 months
Median OS	9.5 months
Grade ≥3 irAEs	6 (25%)
Patients with at least one hospital admission	20 (83.3%)
Total admission episodes	36

Survival outcomes

The median PFS was 4.5 months, and the median OS was 9.5 months. There was considerable variation in PFS across the cohort, with eight patients experiencing disease progression within three months of treatment initiation, while five patients remained progression-free beyond 18 months (Figure 1).

**Figure 1:**
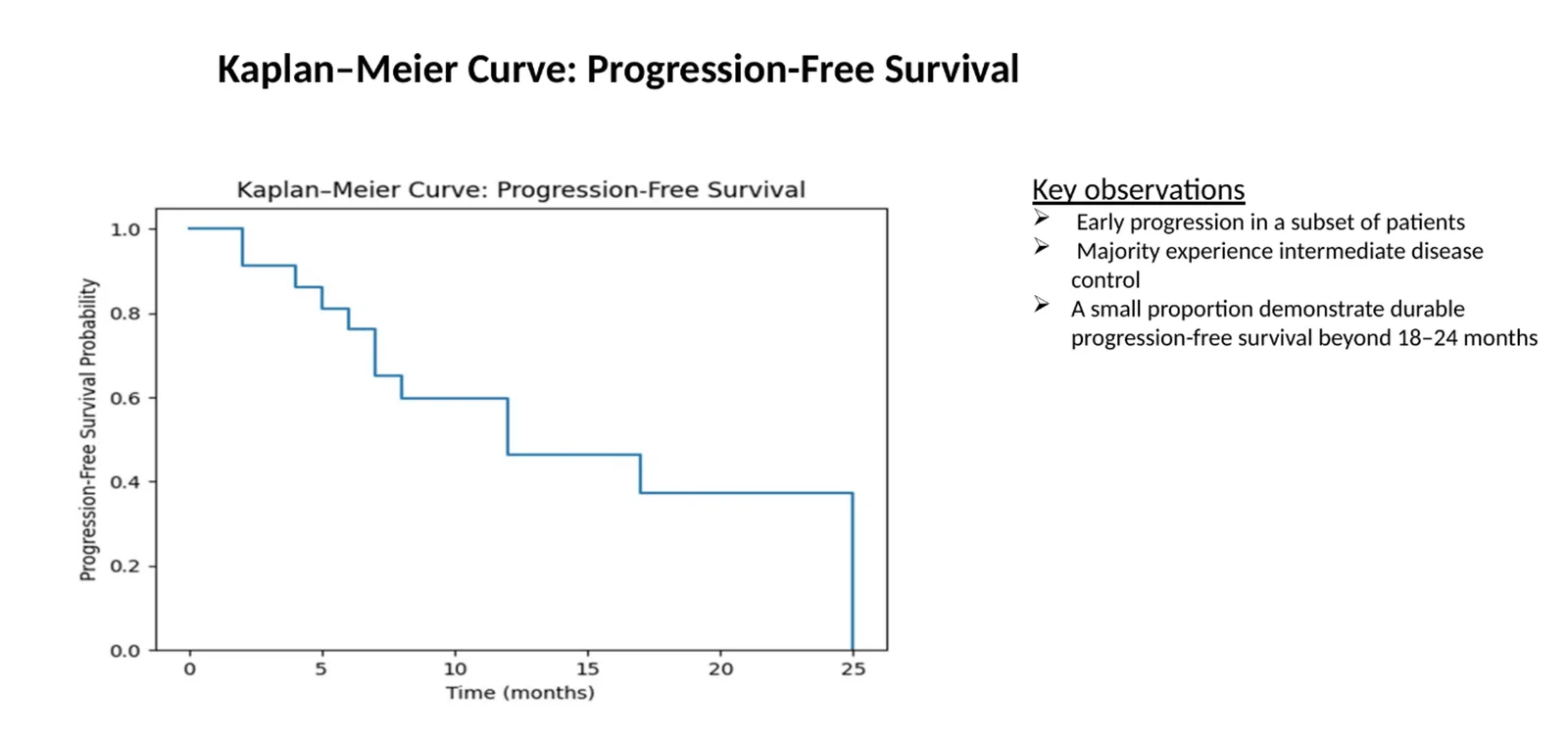
Kaplan-Meier analysis of PFS. Kaplan-Meier analysis demonstrating PFS from initiation of nivolumab and ipilimumab. PFS, progression-free survival

Heterogeneity of treatment outcomes

There was considerable variation in treatment outcomes within this cohort. Eight patients experienced disease progression within three months of initiating treatment, whereas five patients remained progression-free beyond 18 months. This variation highlights the heterogeneity of clinical outcomes following nivolumab and ipilimumab in routine clinical practice. Although baseline performance status, histological subtype, and individual disease characteristics may influence treatment outcomes, a comparison of these factors between patients with early progression and those with prolonged PFS was not possible using the available retrospective data. Therefore, no conclusions can be drawn regarding specific characteristics associated with prolonged disease control in this cohort.

Treatment-related toxicity

Fatigue was the most frequently documented adverse event, affecting 70% of patients, followed by thyroid dysfunction (40%), dyspnea (35%), and elevated hepatic enzymes (20%). Grade 2 or higher toxicity was experienced by 45% of patients, while grade 3 or higher irAEs occurred in six patients (25%). Severe events documented in the cohort included pneumonitis, nephrotic syndrome, heart failure, arthropathy, and hepatitis. However, not all these events were attributed to immunotherapy. Other recorded toxicities involved the gastrointestinal, endocrine, dermatological, renal, and hepatic systems.

Hospital admissions and treatment-related complications

Twenty of the 24 patients (83.3%) experienced at least one hospital admission during or shortly after treatment with nivolumab and ipilimumab, while four patients (16.7%) had no recorded admissions. A total of 36 admission episodes were recorded across the cohort, as some patients experienced multiple admissions. Of the 20 patients admitted, 16 (80%) had admissions attributed to immunotherapy-related adverse effects, while four (20%) had admissions attributed to disease-related or other non-immune complications. No patient was recorded as having both types of admission. Documented immune-related complications included hepatitis, diarrhea, cholangiopathy, nephritis, dermatological toxicity, and hypoadrenalism. Other admission causes included complications associated with advanced malignancy and other medical conditions. The duration of hospital stay varied, ranging from approximately one to 32 days. Management of immune-related toxicities frequently involved corticosteroid treatment, and some adverse events resulted in treatment interruption, discontinuation, or a change in the treatment plan.

## Discussion

Principal findings

This retrospective cohort study provides real-world data on treatment outcomes, toxicity, and hospitalization among patients with advanced mesothelioma treated with nivolumab and ipilimumab. Median PFS was 4.5 months, and median OS was 9.5 months. Although early disease progression was common, a smaller subgroup experienced prolonged disease control, with five patients remaining progression-free beyond 18 months. Treatment exposure also varied considerably, ranging from one to 24 cycles, with a median of five cycles. Hospitalization was also an important finding, with 20 of the 24 patients (83.3%) experiencing at least one admission and 36 admission episodes recorded. These admissions were attributed to both immunotherapy-related adverse effects and disease-related or other non-immune complications.

Comparison with previous evidence

The findings of this study should be interpreted in the context of existing evidence supporting immune checkpoint inhibition in advanced mesothelioma. The phase III CheckMate-743 trial demonstrated improved OS with nivolumab plus ipilimumab compared with platinum-based chemotherapy, establishing dual immunotherapy as an important first-line treatment option for patients with unresectable MPM [3]. Median OS in the CheckMate-743 trial was 18.1 months in the nivolumab plus ipilimumab group, compared with 14.1 months in the chemotherapy group. In our cohort, the median OS was 9.5 months. These differences may reflect variations in patient characteristics, disease burden, performance status, treatment exposure, and follow-up between clinical trial populations and routine clinical practice. This treatment approach is also recognized in clinical practice guidelines [1].

Real-world studies have further evaluated the effectiveness and safety of nivolumab plus ipilimumab in routine clinical practice, providing complementary evidence beyond that generated in controlled clinical trials [4-6]. The survival outcomes observed in the present cohort should be interpreted cautiously alongside these published findings. Differences in patient selection, histological subtype, baseline performance status, treatment duration, follow-up, and management of treatment-related complications may contribute to variations in reported outcomes [4-6]. Furthermore, the small sample size and retrospective design of the present study limit direct comparisons and preclude definitive conclusions regarding treatment effectiveness relative to clinical trial populations or other real-world cohorts.

Toxicity and irAEs

Treatment-related toxicity was an important finding in this cohort. Fatigue, thyroid dysfunction, dyspnea, and elevated hepatic enzymes were among the most frequently documented adverse events. Grade 3 or higher irAEs occurred in 25% of patients, highlighting the potential for clinically significant toxicity associated with dual checkpoint inhibition.

Immune checkpoint inhibitors can cause inflammatory adverse events affecting multiple organ systems, including the gastrointestinal, endocrine, dermatological, renal, pulmonary, and hepatic systems. Early recognition, appropriate severity grading, and timely management are essential components of care for patients receiving these treatments [7]. In the present cohort, adverse events were classified according to their documented clinical attribution. Although severe complications included pneumonitis, nephrotic syndrome, heart failure, arthropathy, and hepatitis, not all were considered immune-related. Some events were attributed to underlying disease, comorbidities, or other clinical complications. This distinction is important when interpreting the toxicity profile, as not all serious events occurring during immunotherapy are necessarily treatment-related.

Corticosteroids were frequently used to manage significant immune-related toxicities in this cohort. Some adverse events resulted in treatment interruption, discontinuation, or modification of the treatment plan. These findings reinforce the importance of systematic monitoring, early recognition, and guideline-based management of irAEs [7].

Hospitalization burden

Hospitalization was common in this cohort, with 20 of 24 patients (83.3%) experiencing at least one hospital admission during or shortly after treatment. Across the cohort, 36 admission episodes were recorded, as some patients experienced multiple admissions. Of the 20 patients admitted, 16 (80%) had admissions attributed to immunotherapy-related adverse effects, while four (20%) had admissions attributed to disease-related or other non-immune complications. No patient was recorded as having both types of admission.

Documented immune-related complications included hepatitis, diarrhea, cholangiopathy, nephritis, dermatological toxicity, and hypoadrenalism. Other admission causes included complications associated with advanced malignancy and other medical conditions, such as pleural effusion, ascites, cardiac failure, gastrointestinal bleeding, sepsis, and procedural or mechanical complications.

The frequency of hospitalization highlights the clinical and healthcare burden associated with managing patients with advanced mesothelioma receiving immunotherapy. Although previous real-world studies have evaluated the safety of nivolumab plus ipilimumab [4-6], direct comparisons of hospitalization rates are limited by differences in study populations, reporting methods, and definitions of treatment-related complications. Our findings emphasize that hospital admissions during immunotherapy may arise from treatment-related toxicity as well as advanced disease and other medical conditions. Careful clinical assessment is therefore essential to establish the underlying cause of admission and guide appropriate management.

Implications for service development: a nurse-led immunotherapy triage pathway

Patients receiving immunotherapy at our institution had access to a 24-hour oncology telephone helpline and specialist nursing support for reporting new symptoms and treatment-related concerns. Despite the availability of these services, 20 patients (83.3%) experienced at least one hospital admission during or shortly after treatment. The present retrospective study did not specifically assess patients’ use of the helpline, the timing of symptom reporting, or the reasons for hospital attendance despite the availability of existing support services.

Clinical implications

These findings highlight the importance of individualized care and multidisciplinary management for patients with advanced mesothelioma receiving nivolumab and ipilimumab. Careful assessment of new or worsening symptoms is essential to distinguish irAEs from complications associated with advanced malignancy or other medical conditions. This distinction can help guide appropriate management and ensure timely intervention throughout treatment.

Strengths and limitations

The main strength of this study is that it provides real-world information on survival, treatment exposure, toxicity, and hospitalization among patients receiving dual immunotherapy for advanced mesothelioma. The inclusion of hospitalization data provides an additional perspective on the clinical burden associated with treatment and advanced disease.

Several limitations should be acknowledged. This was a retrospective study involving a relatively small cohort of 24 patients from a single NHS center, which limits the generalizability of the findings. The cohort included both pleural and peritoneal disease and different histological subtypes. There was also variation in treatment duration between patients.

The study did not include a chemotherapy comparator group. In addition, toxicity and response data were obtained retrospectively from clinical records, and the completeness of documentation may have varied between patients. Although all patients underwent response assessment, the availability of radiological imaging and documentation was not uniform. The retrospective design also limits the ability to establish causality between individual adverse events and immunotherapy. Furthermore, the small sample size and inclusion of patients with different disease sites and histological subtypes mean that the survival and toxicity findings should be interpreted as descriptive and exploratory rather than definitive.

## Conclusions

In this retrospective cohort of 24 patients with advanced mesothelioma treated with nivolumab and ipilimumab, median PFS was 4.5 months and median OS was 9.5 months. Grade 3 or higher irAEs occurred in 25% of patients. Hospital admission was common, with 20 patients (83.3%) experiencing at least one admission and 36 admission episodes recorded.

This study adds to the real-world evidence on dual immunotherapy in advanced mesothelioma by describing survival outcomes, treatment-related toxicities, and hospitalization burden in routine clinical practice. The findings highlight the importance of individualized care, multidisciplinary management, and careful assessment of new symptoms, particularly because hospital admissions may result from immunotherapy-related toxicity as well as disease-related complications. Further prospective evaluation of existing immunotherapy triage pathways may help identify opportunities to improve patient support and coordination of care.
